# Characterizing mild cognitive impairment in prodromal Parkinson’s disease: A community‐based study in China

**DOI:** 10.1111/cns.13766

**Published:** 2021-11-25

**Authors:** Chenxi Pan, Yuqian Li, Jingru Ren, Lanting Li, Peiyu Huang, Pingyi Xu, Li Zhang, Wenbing Zhang, Min‐Ming Zhang, Jiu Chen, Weiguo Liu

**Affiliations:** ^1^ Department of Neurology The Affiliated Brain Hospital of Nanjing Medical University Nanjing China; ^2^ Department of Radiology The Second Affiliated Hospital of Zhejiang University School of Medicine Hangzhou China; ^3^ Department of Neurology The First Affiliated Hospital of Guangzhou Medical University Guangzhou China; ^4^ Department of Geriatrics The Affiliated Brain Hospital of Nanjing Medical University Nanjing China; ^5^ Department of Neurosurgery The Affiliated Brain Hospital of Nanjing Medical University Nanjing China; ^6^ Institute of Brain Functional Imaging Nanjing Medical University Nanjing China; ^7^ Institute of Neuropsychiatry Fourth Clinical College of Nanjing Medical University The Affiliated Brain Hospital of Nanjing Medical University Nanjing China

**Keywords:** cognition, Parkinson’s disease, population‐based study, prodromal

## Abstract

**Objective:**

The International Parkinson and Movement Disorder Society (MDS) has published research criteria for prodromal Parkinson's disease (pPD), which includes cognitive impairment as a prodromal marker. However, the clinical features of mild cognitive impairment (MCI) in pPD remain unknown. Our study aimed to evaluate the frequency and clinical features of mild cognitive impairment of pPD in the elderly in China.

**Methods:**

The cross‐sectional community‐based study recruited 2688 participants aged ≥50 years. Subjects were diagnosed with pPD according to the MDS criteria. Overall, 39 pPD and 22 healthy controls underwent comprehensive clinical and neuropsychological assessment. MCI was also diagnosed by the MDS criteria. Next, we investigated the relationship between clinical factors and cognition.

**Results:**

Among the 2,663 dementia‐free and Parkinson disease (PD)‐free participants, 55 met the criteria for pPD (2.1%) and 23 pPD met the criteria for MCI. Memory, attention/working memory, and executive function were the most frequent impaired domains, and amnestic MCI multidomain phenotype was the most frequent MCI subtype (69.57%) in pPD. Additionally, correlation analysis revealed that the global cognitive performance was negatively related to UPDRS‐III score (*r* = −0.456, *p* = 0.004).

**Conclusion:**

MCI, specifically impairment in memory, attention/working memory, and executive domain, is present at the prodromal stage of PD. In addition, cognitive performance is correlated with motor symptoms in pPD. Our results reflect that cognitive profile, combined with motor symptoms, can help clinicians to identify individuals with pPD early, as those would be the optimal candidates for neuroprotective therapy.

## Graphical abstract

Mild cognitive impairment, specifically impairment in memory, attention/working memory, and executive, is present in prodromal PD in an elderly population of China. In addition, cognitive performance is correlated with motor symptoms in prodromal PD. The results reflect that cognitive profile, combined with motor symptoms, can help clinicians to identify individuals with prodromal PD early, as those would be the optimal candidates for neuroprotective therapy.

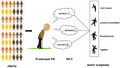

## 1 INTRODUCTION

Parkinson's disease (PD) is a neurodegenerative disorder and whose diagnosis criteria include motor symptoms as the core feature of the disease. The motor symptoms are defined as bradykinesia, rest tremor, or rigidity.^1^ Several studies demonstrated that 40%–60% of dopaminergic neurons had already degenerated by the time motor symptoms met the criteria for PD.^2^, ^3^, ^4^ The neurodegenerative period, when non‐motor symptoms or mild parkinsonian signs are present, without the classical motor symptoms, is defined as prodromal PD (pPD).^5^ In addition, PD is becoming strongly recognized as a multisystemic disorder with both motor and non‐motor symptoms, and certain non‐motor features present decades prior to achieving a clinical diagnosis.^6^

One of the key points of the current research in PD is the identification and characterization of its prodromal period. Prior longitudinal studies, including large population‐based cohort, have investigated the risk factors for pPD.^7^, ^8^, ^9^ Unfortunately, studies in this field are limited, as longitudinal study is time‐consuming, costly, and poor timeliness. Besides, due to the low incidence rate of PD and different definitions of pPD, some studies have focused on Rapid eye movement (REM) sleep behavior disorder (RBD) patients and individuals with different genetic mutations who are subsequently diagnosed with PD, but whose features may differ from individuals with typical sporadic PD.^10^, ^11^, ^12^ Furthermore, based upon non‐motor markers, the International Parkinson and Movement Disorder Society (MDS) published research criteria for pPD,^5^ with high specificity.^13^ Thus, in order to better understand the risk factors of PD in East China population, our group established a prospective, community population‐based pPD cohort in Jiangsu province.

It is interesting to note that the percentage of memory decline was high in the pPD group, based on our preliminary research. Mild cognitive impairment (MCI), an intermediate state between normal cognitive aging and early dementia,^14^ was present in newly diagnosed PD patients.^15^, ^16^ In a retrospective study, 5.5% of PD patients complained about experiencing non‐specific “cognitive impairment” prior to their diagnosis.^17^ In addition, previous studies have suggested that cognitive impairment is related to incidence of future PD.^7^, ^8^, ^18^, ^19^ Meanwhile, the MDS recently updated the diagnosis of pPD, and added global cognitive defect as a prodromal marker.^20^, ^21^ However, the majority of studies, which use questionnaires or limited cognitive tests, have failed to evaluate the clinical characteristics of MCI among individuals that are at risk for PD in the general elderly population.^7^, ^17^, ^22^

The objectives of this present study were to (1) investigate the individuals presumed to be at an increased risk of PD in a community‐dwelling elderly population of East China, and (2) to examine the cognitive profile of individuals with prodromal PD. We hope that our study is able to provide important proof to investigate the features of individuals with pPD in a community‐dwelling elderly population of East China.

## 2 METHODS

### 2.1 Study population

**FIGURE 1:**
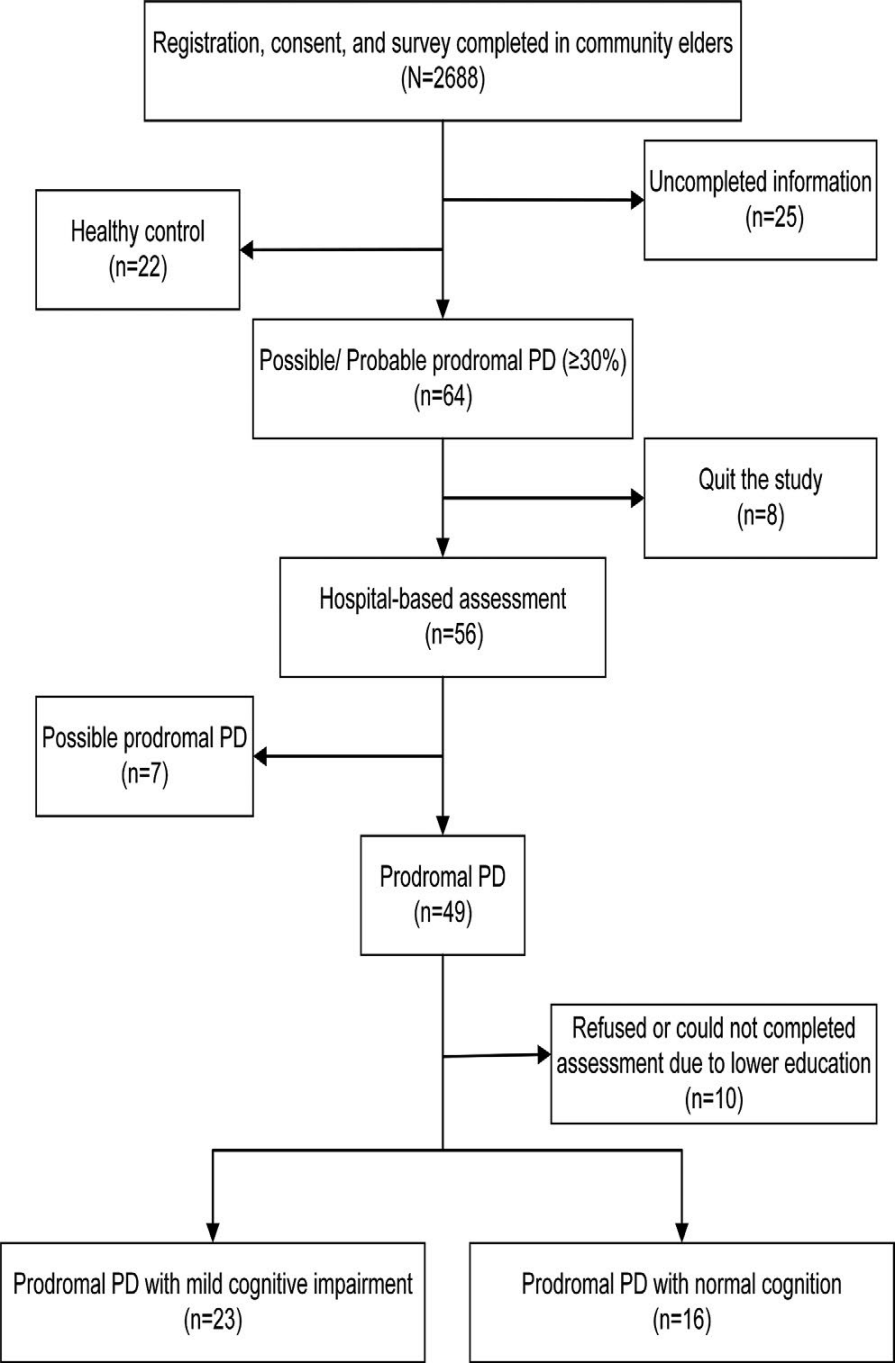
The flow of participants in the study. Abbreviations: pPD, prodromal Parkinson's disease

This study recruited 2,688 volunteers from approximately nine districts across Nanjing City between March 2017 and August 2020. Overall, 25 volunteers were excluded due to incomplete information, and 2663 participants comprised the sample of the community survey. We found 64 possible/probable prodromal PD patients (i.e., ≥30% probability of pPD) in the community survey. Next, we invited them to the hospital by telephone for further examination. However, two possible pPD and six probable pPD participants quit the study. In further hospital‐based assessment, seven participants were diagnosed as possible pPD, and 49 were diagnosed as probable pPD. But 10 of probable pPD refused or had insufficient knowledge to carry out neuropsychological assessment. Finally, 39 pPD (i.e., probable pPD) were divided into either a pPD group with mild cognitive impairment (pPD‐MCI) or a pPD group with normal cognition (pPD‐NC) (See Figure 1). Inclusion criteria in this study were residents who had lived in the local community for longer than 1 year and between the age of 50–80 years. Exclusion criteria were as follows: (1) pre‐existing PD, Parkinsonism, or other movement disorder; (2) dementia; (3) history of serious diseases, such as cancer, hyperthyroidism, severe psychiatric, and systemic illnesses.

For further analyses, we recruited 22 healthy controls (HCs). The inclusion criteria for HCs were as follows: (1) post‐test probability of pPD <30%; (2) normal cognitive performance of age‐ and education‐matched volunteers, and (3) at least a primary school education. The exclusion criteria for HCs included: (1) history of serious diseases, such as cancer, diabetes, hyperthyroidism, severe psychiatric, and systemic illnesses; (2) pre‐existing neurodegenerative diseases; and (3) prescription of certain medications, such as antidepressant drugs.

### 2.2 Standard protocol approvals, registrations, and patient consents

The study was granted approval by the Medical Ethics Committee of the Second Affiliated Hospital of Zhejiang University College of Medical and the Affiliated Brain Hospital of Nanjing Medical University. All participants were required to sign informed consent prior to participation.

### 2.3 MDS research criteria and selection of markers

The MDS defined possible and probable pPD as having a probability score of 30%–80% and ≥80%, respectively.^5^, ^23^ Additionally, the MDS considered probable pPD met the criteria for prodromal PD (pPD).^5^

We calculated the probability of pPD using available markers, as suggested by MDS and conducted by prior studies.^20^, ^24^ Several risk markers were used for the calculation, including male sex, occupational solvent exposure, regular pesticide exposure, coffee or tea use, non‐smoking status, family history (including siblings with PD at age onset <50 years old or any first‐degree relative with PD), and abnormal hyperechogenicity of the substantia nigra (SN). Additional prodromal markers were also assessed, including possible subthreshold parkinsonism, RBD (Polysomnographic‐proven idiopathic RBD or positive RBD screen questionnaire), constipation, olfactory loss, excessive daytime somnolence, symptomatic hypotension, urinary dysfunction, severe erectile dysfunction, and depression/anxiety.

### 2.4 Community‐based assessment

All participants underwent a face‐to‐face interview, and all investigators that carried out the questionnaire received questionnaire application training. We collected socio‐demographic information (i.e., gender, age, education levels, alcohol and tea use, and leisure activities) of the participants. Diagnosis of prior diseases (e.g., diabetes, hypertension, hyperlipidemia, stroke, coronary heart disease, and mood disorders) was obtained via self‐report. We utilized a standardized structured questionnaire to evaluate the presence of a variety of environmental and lifestyle risk markers, as well as prodromal markers, during the initial interview. Standardized structured questionnaire, which included risk and prodromal markers, was obtained via self‐report, and current use of medications (i.e., calcium channel blockers, beta‐blockers, statins, antiplatelets, antidepressants, and benzodiazepines) were also recorded.

### 2.5 Hospital‐based assessment

Overall, 56 individuals with possible/probable pPD underwent a standardized interview and neurologic examination, which was performed by two specialized neurologists. Medical history, medication history, family history focused on movement disorders, and possible causes for secondary parkinsonism were all recorded. The Unified Parkinson's Disease Rating Scale motor section (UPDRS‐III) was utilized to assess motor function, the Non‐Motor Symptoms Scale (NMSQ) was used to evaluate non‐motor symptoms, and the Mini Mental State Examination (MMSE) and the Montreal Cognitive Assessment (MOCA) were utilized to evaluate cognition. Depression and anxiety were defined through the use of the Hamilton Anxiety Scale (HAMA) and the Hamilton Depression Rating Scale (HAMD). Clinical RBD symptoms were determined using the REM sleep behavior disorder questionnaire‐Hong Kong (RBDQ‐HK)^25^ or Polysomnography (PSG). Furthermore, we utilized the Sniffin’ Sticks test^26^ to screen possible/probable pPD patients for olfactory dysfunction, and SN ultrasound to screen for abnormal hyperechogenicity of the SN.

### 2.6 MCI diagnosis and cognitive status assessment

Mild cognitive impairment was diagnosed as per the MDS Task Force Level II diagnostic criteria, which provides an optimal, efficient neuropsychological test battery for diagnosis,^27^ and applied assessment of the MCI subtypes.^15^, ^28^ The pPD and HC subjects were administered a formal, comprehensive neuropsychological battery. The neuropsychological battery, including memory, visuospatial function, language, attention/working memory, and executive function, was performed with the following tests: Auditory Verbal Learning Test (AVLT), Logical Memory Test (LMT), Benton's Judgment of Line Orientation Test (JLOT), Hooper Visual Organization Test (HVOT), Boston Naming Test (BNT), Wechsler Adult Intelligence Scale III (WAIS‐III) Similarities Test, Digit Span Backward Test (DST), Trail Making Test A (TMT‐A), Stroop Color‐Word Test (SCWT), Trail Making Test B (TMT‐B), Clock Drawing Test (CDT), and Verbal Fluency Test (VFT).

### 2.7 Statistical analyses

The data were analyzed by using the Statistical Package for the Social Sciences (SPSS) statistical software package (version 25). The demographic and clinical continuous variables are represented as mean ± standard deviation (SD). The normality assumption of data was assessed by using the Kolmogorov‐Smirnov test (*n* > 50) or Shapiro‐Wilk test (*n* ≤ 50). The differences of continuous variables with a normal distribution among the three groups (pPD‐MCI, pPD‐NC, HC) were analyzed by the one‐way analysis of variance (ANOVA), and the continuous variables that do not exhibit a normal distribution were analyzed by the Kruskal‐Wallis test. According to the normality of distribution and homogeneity of continuous variance, the Bonferroni or Games‐Howell test was utilized after ANOVA and the Bonferroni test was used after Kruskal‐Wallis test for multiple comparisons. Binominal variables are represented as the number and percentage of their respective category. The differences of categorical variables among the three groups were analyzed by using the Chi‐squared test. We set *p* < 0.05 as the threshold for statistical significance.

As previously described,^18^, ^29^ we transformed scores from cognitive tests into z scores using mean and SD values of the HC group. Next, a domain z score was calculated by averaging individual neuropsychological test z scores (Table 1 shows domains and corresponding neuropsychological tests). A global z score based on the domain z scores was calculated in the same manner. For analytical purposes, the scores of some cognitive tests were reversed, and a higher global z score indicates better cognitive performance.

**TABLE 1:** Demographics and clinical characteristics of the pPD groups and healthy control group

	pPD‐MCI (*n* = 23)	pPD‐NC (*n* = 16)	HC (*n* = 22)	*p*
Age (year)	65.74 ± 4.67	66.94 ± 8.54	64.23 ± 5.32	0.398^a^
Gender (M/F)	9/14	7/9	12/10	0.572^b^
Education (y)	9.09 ± 2.64^‡*, §*^	11.69 ± 3.18	11.34 ± 2.83	0.009^a^
UPDRS‐Ⅲ score	10.70 ± 4.76§^***^	7.06 ± 6.13^¶***^	0.59 ± 1.62	0.000^c^
NMSQ	12.00 ± 5.49^§***^	11.06 ± 4.46^¶***^	1.55 ± 1.90	0.000^c^
HAMA	8.26 ± 5.41^§***^	6.56 ± 4.60^¶***^	0.50 ± 1.19	0.000^c^
HAMD	11.22 ± 7.73^§***^	9.13 ± 6.46^¶***^	0.77 ± 1.97	0.000^c^
RBDQ‐HK	26.70 ± 15.67^§***^	30.75 ± 13.89^¶***^	6.27 ± 6.01	0.000^a^
MMSE	26.87 ± 2.01^‡*, §*^	28.50 ± 1.71	28.36 ± 1.53	0.008^c^
MOCA	21.61 ± 2.64^‡**, §***^	24.94 ± 2.24	26.82 ± 2.34	0.000^c^
Attention/Working memory				
DST	10.83 ± 2.35	12.25 ± 2.02	11.95 ± 2.38	0.114^c^
TMT‐A (s)	108.91 ± 38.41^‡**, §**^	76.75 ± 28.34	76.68 ± 23.36	0.000^c^
SCWT‐C‐ right	47.19 ± 3.86^#^	48.44 ± 2.76	47.50 ± 2.87	0.286^c^
Executive				
TMT‐B (s)	198.71 ± 56.35^‡*, §*^	159.44 ± 41.38	160.91 ± 30.85	0.009^a^
CDT	9.22 ± 1.17	9.38 ± 1.20	9.82 ± 0.59	0.121^c^
VFT	17.09 ± 4.31^‡*^	20.69 ± 5.20	18.77 ± 2.99	0.035^a^
Memory				
AVLT‐delayed recall	3.22 ± 1.98^‡*, §***^	5.13 ± 2.19^¶*^	7.05 ± 2.66	0.000^a^
LMT‐delayed recall	4.73 ± 2.55^‡**, §***^	7.50 ± 1.67	6.91 ± 2.32	0.001^c^
Visuospatial function				
JLOT	23.46 ± 3.11	25.56 ± 2.73	25.55 ± 2.36	0.024^c^
HVOT	13.20 ± 4.12^‡*, §*^	16.59 ± 4.27	16.50 ± 3.31	0.008^a^
Language				
Similarities	14.70 ± 4.27^§*^	17.31 ± 3.63	16.95 ± 3.80	0.107^c^
BNT	21.61 ± 4.21^‡*, §**^	24.94 ± 2.14	25.14 ± 2.88	0.003^c^

Note

Data are presented as mean ± SD. The results of post hoc multiple comparisons (Bonferroni or Games‐Howell for one‐way ANOVA, Bonferroni for Kruskal‐Wallis test) were indicated as: ^‡^MCI‐NC; ^§^MCI‐HC; ^¶^NC‐HC.

Abbreviations: AVLT, Auditory Verbal Learning Test; BNT, Boston Naming Test; CDT, Clock Drawing Test; DST, Digit Span Backward Test; F, female; HAMA, Hamilton Anxiety Scale; HAMD, Hamilton Depression Rating Scale; HVOT, Hooper Visual Organization Test; JLOT, Benton's Judgment of Line Orientation Test; LMT, Logical Memory Test; M, male; MMSE, Mini Mental State Examination; MOCA, Montreal Cognitive Assessment; NMSQ, Non‐Motor Symptoms Scale; pPD‐MCI, prodromal Parkinson's disease with mild cognitive impairment; pPD‐NC, prodromal Parkinson's disease with normal cognitive; RBDQ‐HK, REM sleep behavior disorder questionnaire‐Hong Kong; SCWT‐C, Stroop Color‐Word Test task 3(Card C); TMT‐A, Trail Making Test A; TMT‐B, Trail Making Test B; UPDRS‐III, Unified Parkinson's Disease Rating Scale motor section; VFT, Verbal Fluency Test; y, year.

*p < 0.05.

**p < 0.01.

***p < 0.001.

^a^ One‐way ANOVA.

^b^ Chi‐square test.

^c^ Kruskal‐Wallis test.

Logistic regression analyses (dichotomous variable) were conducted in order to explore the influence of potential confounding factors, including sex, the use of non‐steroidal anti‐inflammatory drugs (NSAID), and memory decline, on possible/probable pPD. Partial correlation analysis (for normally distributed data) was performed to investigate the relationship between global cognition and clinical features in pPD, controlling the effects of age, years of education, gender, and HAMD.

## 3 RESULTS

**FIGURE 2:**
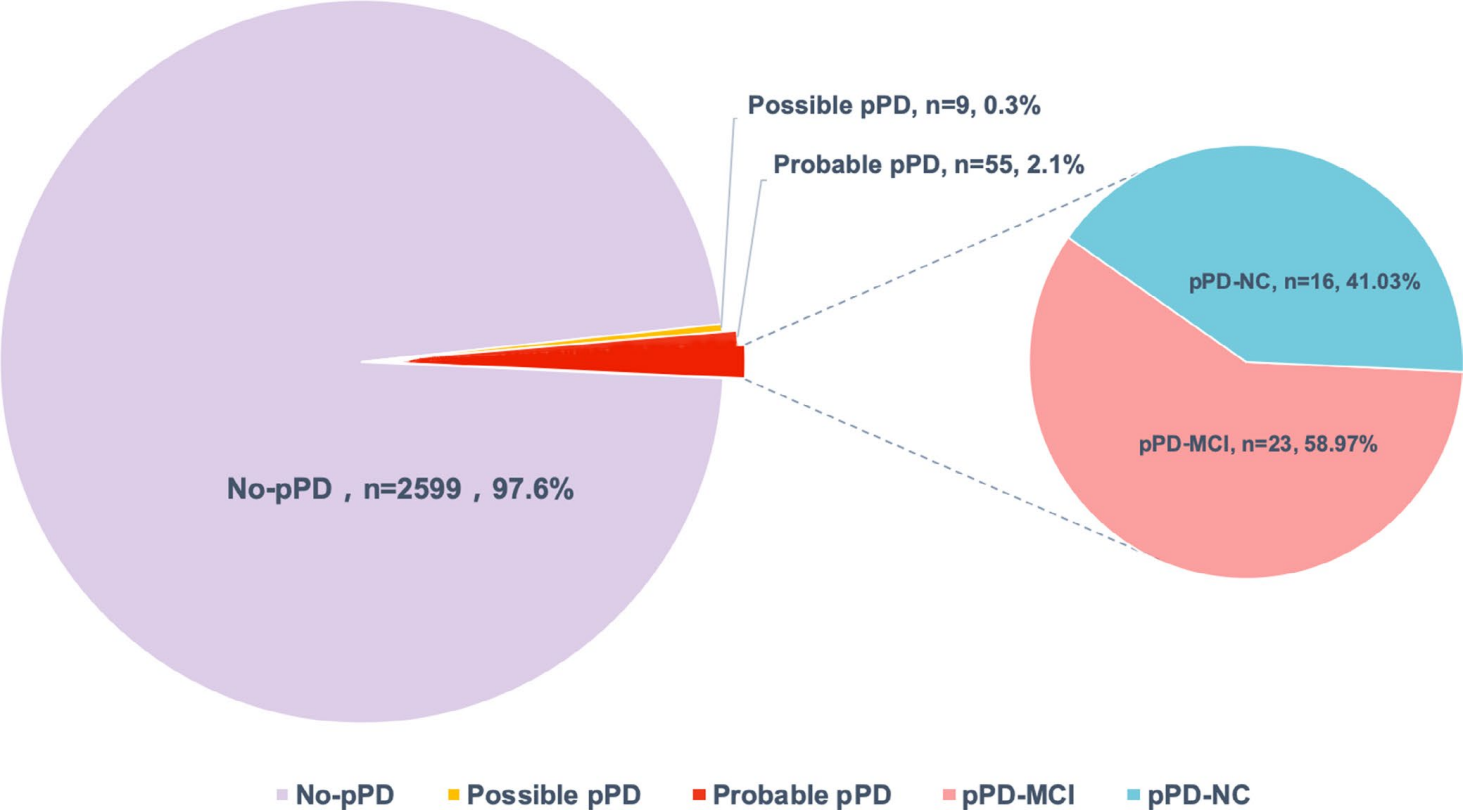
Pie chart reflecting the proportion of different groups in its whole samples. Abbreviations: pPD, prodromal Parkinson's disease; pPD‐MCI, prodromal Parkinson's disease with mild cognitive impairment; pPD‐NC, prodromal Parkinson's disease with normal cognitive

The flow of participants in the study are shown in Figure 1. Among the 2,663 dementia‐free and PD‐free participants, 64 participants met the criteria for possible/probable pPD (prevalence, 2.4%; 95% CI, 1.8%–3.0%). Furthermore, 55 participants met the criteria for probable pPD (prevalence of 2.1%; 95% CI, 1.5%–2.6%) (Figure 2). Characteristics of the 2,663 subjects in the community population are detailed in Table 2. The proportion of females in possible/probable pPD group is lower compared with the other group, while the percentage of memory decline and NSAID use in possible/probable pPD group is higher compared with non‐pPD group. Furthermore, the percentage of memory decline is noticeably high in possible/probable pPD group. As expected, logistic regression analysis demonstrated that compared with those with less than 30% probability score for pPD, those with possible/probable pPD are three‐fold more likely to experience memory decline (OR 3.372; 95% CI 1.709–6.651; *p* < 0.001).

**TABLE 2:** Demographics and clinical characteristics of the community study population

	All participants (*n* = 2,663)	Non‐pPD group pPD probability <30% (*n* = 2599)	pPD group pPD probability ≥30% (*n* = 64)
Age (year)^a^	66.2 ± 5.7	66.2 ± 5.7	65.0 ± 6.9
Female (%)^b^	1635 (61.4%)	1599 (60.1%)	36 (56.2%)
Family history of PD^b^	95 (3.6%)	25 (1.0%)	10 (15.6%)
Regular pesticide exposure^b^	662 (24.9%)	639 (24.6%)	23 (35.9%)
Occupational solvent exposure^b^	652 (24.5%)	628 (24.2%)	24 (37.5%)
Smoker^b^			
Current	584 (21.9%)	568 (21.9%)	16 (25.0%)
Former	30 (1.1%)	30 (1.1%)	0 (0.0%)
Never	2049 (77.0%)	2001 (77.0%)	48 (75.0%)
Coffee use^b^	248 (9.1%)	239 (9.2%)	17 (7.1%)
Tea use^b^	1153 (43.3%)	1134 (43.6%)	19 (29.7%)
Alcohol^b^	573 (21.5%)	559 (21.5%)	14 (21.9%)
Olfactory loss^b^	168 (6.3%)	144 (5.5%)	24 (37.5%)
Constipation^b^	387 (14.5%)	360 (13.9%)	27 (42.2%)
Excessive daytime somnolence^b^	202 (7.6%)	183 (7.0%)	19 (29.7%)
Symptomatic hypotension^b^	818 (30.7%)	785 (30.2%)	33 (51.6%)
Severe erectile dysfunction (man)^b^	6 (0.6%)	5 (0.2%)	1 (1.6%)
Urinary dysfunction^b^	467 (17.5%)	447 (17.2%)	20 (31.3%)
Depression^b^	366 (9.8%)	334 (12.9%)	32 (50.0%)
NSAID use^b^	328 (12.3%)	318 (12.2%)	10 (15.6%)
Memory decline^b^	1654 (62.1%)	1600 (61.6%)	54 (84.4%)

Abbreviations: CCB, calcium channel blocker; NSAID, non‐steroidal anti‐inflammatory drugs; PD, Parkinson's disease; pPD, prodromal Parkinson's disease.

^a^ Quantitative results are reported in mean ± SD.

^b^ Binominal variables are given in number and percentage of the respective category.

**FIGURE 3:**
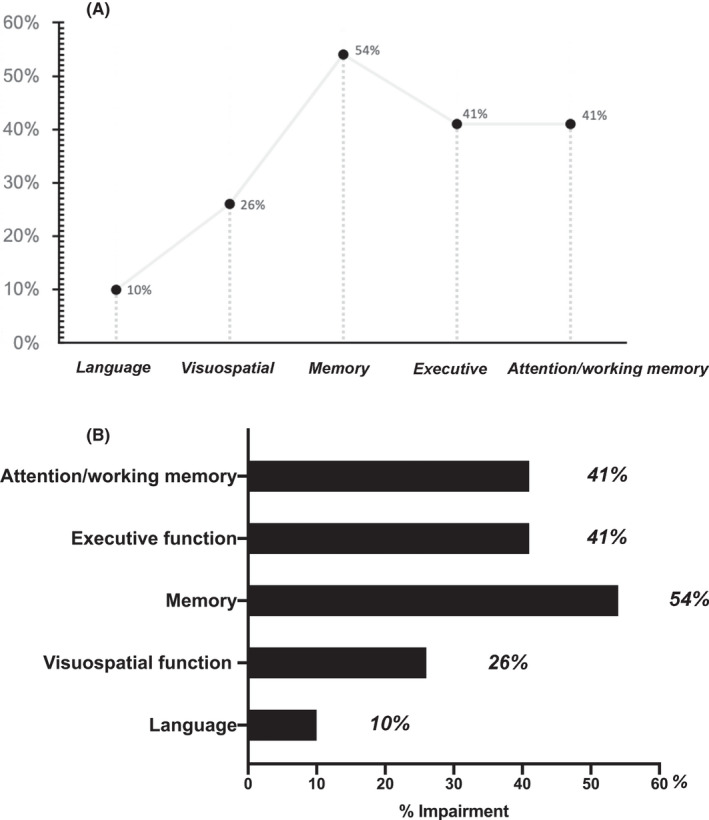
The frequency of each abnormal cognitive domain in the whole prodromal Parkinson's disease population

Compared to the HC group, subjects in the pPD group performed worse in all cognitive tests, with the greatest differences for TMT‐A, AVLT‐delayed recall, and BNT scores (See Table S1). Among the 39 pPD, 23 fulfilled the diagnosis of MCI (58.97%; 95% CI, 43.79%–74.15%) (Figure 2). The most common subtype included amnestic MCI multi‐domain (aMCImd; 69.57%), which was followed by non‐amnestic MCI multiple domain (naMCImd; 17.39%) and amnestic MCI single domain (aMCIsd; 13.04%). On the other hand, no case of non‐amnestic MCI single domain (naMCIsd) was recorded. Moreover, considering the presence of at least one impaired test, the domain that was most frequently affected among the pPD group was memory (54%), followed by attention/working memory function (41%), executive function (41%), visuospatial (26%), and language (10%) (Figure 3). Demographics and clinical characteristics of pPD and HCs (*n* = 22) are presented in Table 2. Compared to the HC group and pPD‐NC group, the pPD‐MCI group had significantly poorer results of the TMT‐A, TMT‐B, AVLT‐delayed recall, LMT‐delayed recall, HVOT, and BNT tests (Table 1).

The global z score was negatively correlated with UPDRS‐III score (*r* = −0.456, *p* = 0.004) among individual with pPD, after adjusting for the effects of age, years of education, gender, and HAMD.

## 4 DISCUSSION

To date, this is one of largest general community cross‐sectional studies taken in the east of China. Through by the use of recently published MDS research criteria for pPD, we set out to identify a group of individuals with risk factors of developing PD and investigated the cognitive changes in pPD. We found that the prevalence of possible/probable pPD and probable pPD in general elderly population was 2.4% and 2.1%, respectively. Additionally, 58.97% of the pPD population fulfilled the diagnosis of MCI, and better cognitive performance is correlated with lower motor scores. Considering the different domains throughout the pPD group, the most frequently impaired domain was memory, and then followed by attention/working memory and executive function.

According to our study, the cognitive profile of prodromal PD was characterized by changes that occur most frequently in memory function. The Prospective Evaluation of Risk Factors for Idiopathic Parkinson's Syndrome (PRIPS) study demonstrated that memory of future PD converters was significantly impaired.^30^ Our finding suggests that attention/working memory and executive function are the second most frequently affected cognitive domain. The Rotterdam Study identified that PD converters performed significantly worse on executive function and memory prior to diagnosis.^8^ In a longitudinal population‐based Honolulu‐Asia‐Aging Study (HAAS) study, lower executive performance was related to an increased risk of incident PD.^7^ In addition, changes were pronounced on executive function/working memory, memory, and attention/processing speed among individuals that were at risk for PD in the Parkinson‐Associated Risk Syndrome (PARS) study.^29^ Notably, knowledge on cognitive domain changes in previous pPD study was different, likely due to diverse definition for prodromal PD, small samples, and a limited number of studies.

In order to better understand whether different cognitive domain impairments have different neurobiological basis or not, classification of MCI subtypes is important.^28^ A higher frequency of the multiple‐domain MCI subtype (86.96%) compared with the single domain (13.04%) was found, which concerns prevalence of specific MCI subtypes. The most common MCI subtype was aMCImd (69.57%), followed by naMCImd (17.39%), aMCIsd (13.04%). To the best of our knowledge, only one prospective RBD study reported the prevalence of specific MCI subtypes.^31^ However, probable PD in this study was based on RBD patients instead of general population, and the sample was small (only eight patients), and only three cognitive domains were assessed. Thus, the results need to be interpreted with caution. In addition, a previous study reported higher frequency of multi‐domain MCI subtypes versus the single domain in newly diagnosed PD patients,^15^ thereby confirming our results.

Although our results do not support any firm conclusions with regard to the etiology of pPD‐MCI, certain previous studies are able to explain these findings. On one hand, the relationship between cognitive and motor functions was found among early untreated PD patients, as well as participants with mild parkinsonian signs (MPS), which may point to overlapping dopaminergic network systems or pathologies.^32^, ^33^ In one pathological study, there was a remarkable reduction in dopaminergic neurons and terminals within the substantia nigra and putamen among subjects with minimal motor symptoms, who may represent pPD.^34^ Chahine et al.^35^ also considered that cognitive dysfunction among individuals at an increased risk for PD seems to be caused by neurodegeneration of the dopamine system. On the other hand, impairment of noradrenergic and cholinergic systems occurs early, according to Braak's staging hypothesis, prior to the involvement of nigral or cortical neurons, and may be partially involved in the pathological process of cognitive decline among individuals with pPD.^36^ However, Burke et al.^37^ considered the relationship between the early Braak stage of abnormal synuclein staining and PD to be uncertain. Chahine et al.^29^ even suggested that cortical involvement with Lewy body pathology may be earlier compared to nigral pathology in parts of patients with prodromal PD. Thus, as the model proposes,^38^, ^39^ both the limbic cortex and lower brainstem are involved in cognitive changes within the prodromal stage of PD.

Furthermore, very few studies have evaluated the prevalence of individuals that are at risk of PD in the community‐based population, using recent MDS criteria. The longitudinal population‐based Bruneck Study demonstrated that the prevalence rate of probable prodromal PD in 539 participants was 2.2%.^24^ Among the Hellenic Longitudinal Investigation of Aging and Diet (HELIAD) study, 38 of 1629 participants met the diagnosis of possible/probable pPD criteria (2.3%).^18^ In this regard, our data suggest that the prevalence of pPD in the community‐based elderly population of East China is consistent with that of other population.

Our study also has limitations. Firstly, it was a single‐center cross‐sectional study that did not allow us to attribute a causal relationship. Further multi‐center longitudinal studies with large sample sizes and longer follow‐up need to be carried out to determine whether pPD converts in PD or not. Additionally, it will allow us to track the dynamic changes of cognitive performance in prodromal PD individuals, which will permit us to determine whether MCI can be used to predict the likelihood of PD conversion from pPD. At present, cohorts from other centers have been established in succession, and our center has been performing regular follow‐ups. Secondly, the current research was a pilot exploratory study, and neuroimaging data were not available in it. Recent studies have found that atrophy in frontal and temporal lobes, and changes of brain structural network were detected at prodromal stage of PD or in PD‐NC patients.^40^, ^41^ Besides, reduced cortical cerebral blood flow (CBF) was found in preclinical PD mice model and prodromal AD participants.^42^, ^43^ Thus, neuroimaging techniques have become useful tools to research the functional and structure changes of brain in prodromal PD. Future studies with neuroimaging data would be able to confirm these findings and explore the neural bases. Finally, the pPD criteria recommended by MDS are based upon the probability that prodromal disease is present. Indeed, the MDS criteria allocated pPD as a high likelihood (≥80%), and in the longitudinal cohort study it has been suggested to be a promising tool to identify incident PD.^24^

## 5 CONCLUSION

Taken together, this present study investigates the prevalence of pPD in an elderly community‐based population of China and confirms cognitive dysfunction as a risk factor for the development of PD. Additionally, half of the pPD cohort demonstrated an MCI phenotype at diagnosis and were more likely to report multiple‐domain MCI subtypes. The highest frequency of impaired domain in subjects with pPD was memory, followed by attention/working memory and executive function. Future longitudinal studies are warranted to assess cognitive symptoms with multiple time points and to determine the most sensitive cognitive domains leading to PD conversion. Although prospective studies are warranted, acknowledging the possible relationship of cognition and UPDRS‐III score in prodromal PD can alert clinicians to look for motor symptoms in MCI patients, as well as for relevant non‐motor symptoms and inform the probability of future PD. Furthermore, if possible, it can provide clues on how or when we may be able to intervene with neuroprotective or disease‐modifying therapies.

## CONFLICT OF INTEREST

The author declares that there is no conflict of interest.

## AUTHOR CONTRIBUTIONS

WGL, PYH, MMZ, LZ, WBZ, and PYX made substantial contributions to the conception or design of the work. CXP, YQL, JRR, LTL, and WGL made substantial contributions to the acquisition of the data. CXP, YQL, JC, and WGL made substantial contributions to the analysis of the data. CXP, JC, and WGL made substantial contributions to the interpretation of the data. CXP drafted the work.

## Supporting information

Table S1

## Data Availability

The data that support the findings of this study are available from the corresponding author upon reasonable request.
