# Supplementary material for: Characterizing mild cognitive impairment in prodromal Parkinson’s disease: A community‐based study in China
Source: CNS Neurosci Ther. 2021 Nov 25;28(2):259–68. doi: 10.1111/cns.13766 (PMC8739042; doi:10.1111/cns.13766)
Supplement: Supplementary file 1 — Table S1 [file CNS-28-259-s001.doc]

**Supplementary Materials**

Table 1. Cognitive characteristics of the pPD and healthy control groups.

|  | pPD（n = 39） | HC（n = 22） | P |  |
| --- | --- | --- | --- | --- |
| MMSE | 27.54 ± 2.04 | 28.36 ± 1.53 | 0.132 ^‡^ |  |
| MOCA | 22.97 ± 2.96 | 26.82 ± 2.34 | 0.000 ^†^ |  |
| Attention/Working memory | |  |  |  |
| DST  TMT-A(s)  SCWT-C-right | 11.41 ± 2.30  95.72 ± 37.79  47.73 ± 3.44 | 11.95 ± 2.38  76.68 ± 23.36  47.50 ± 2.87 | 0.404 ^‡^  0.030 ^‡^  0.308 ^‡^ |  |
| Executive  TMT-B(s)  CDT  VFT | 181.73 ± 53.54  9.28 ± 1.17  18.56 ± 4.96 | 160.91 ± 30.85  9.82 ± 0.59  18.77 ± 2.99 | 0.210 ^‡^  0.051 ^‡^  0.460 ^‡^ |  |
| Memory  AVLT-delayed recall  LMT-delayed recall | 4.00 ± 2.25  5.90 ± 2.60 | 7.05 ± 2.66  6.91 ± 2.32 | 0.000 ^‡^  0.135 ^†^ |  |
| Visuospatial function | |  |  |  |
| JLOT  HVOT | 24.32 ± 3.10  14.59 ± 4.46 | 25.55 ± 2.36  16.50 ± 3.31 | 0.170 ^‡^  0.085 ^†^ |  |
| Language  similarities  BNT | 15.77 ± 4.18  22.97 ± 3.85 | 16.95 ± 3.80  25.14 ± 2.88 | 0.271 ^‡^  0.019 ^‡^ |  |

Data are presented as mean ± SD.

^†^ parametric test (Student t test).

^‡^ nonparametric test (Mann-Whitney U test).

Abbreviations: pPD, prodromal Parkinson’s disease; HC, healthy control; MMSE, Mini Mental State Examination; MOCA, Montreal Cognitive Assessment; DST, Digit Span Backward Test; TMT-A, Trail Making Test A; s second; SCWT-C, Stroop Color-Word Test task 3(Card C); TMT-B, Trail Making Test B; CDT, Clock Drawing Test; VFT, Verbal Fluency Test; AVLT, Auditory Verbal Learning Test; LMT, Logical Memory Test; JLOT, Benton’s Judgment of Line Orientation Test; HVOT, Hooper Visual Organization Test; BNT, Boston Naming Test.
